# Supplementary material for: Using somatic variant richness to mine signals from rare variants in the cancer genome
Source: Nat Commun. 2019 Dec 3;10:5506. doi: 10.1038/s41467-019-13402-z (PMC6890761; doi:10.1038/s41467-019-13402-z)
Supplement: Supplementary file 1 — Supplementary Information [file 41467_2019_13402_MOESM1_ESM.pdf]

Supplementary Information for "Using Somatic Variant Richness to Mine Signals from Rare Variants in the Cancer Genome" by Chakraborty et al.

Supplementary Methods

Given a training sequencing cohort of m tumors, we seek to estimate the probabilities associated with encountering variants – those previously observed in the training sample, and those hitherto unseen – in a new tumor outside the training sample. To accomplish this, we extend and make use of the Good-Turing strategy described immediately below. The probability of encountering a previously seen variant can be readily estimated using this method, as can the probability of observing *at least one* hitherto unseen variant. These methods can then be applied to estimate the probabilities associated with encountering specific variants and the number of unseen variants in the entire cancer genome, and also at the gene specific level when considering a single gene as a particular sampling frame of interest. We note that we have restricted our analyses to non-synonymous single nucleotide variants, but the methods described in the following could be applied to silent mutations or more complex somatic variants.

We denote by q_j and y_j respectively, the probability of encountering the j-th variant in a randomly selected tumor, and the number of times that variant appears in the existing sample of m tumors, $j = 1, \ldots, N$, where N is the total number of variants. Then $y_j \sim$ Binomial(m, q_j), and assuming independence among the occurrences of variants, we obtain

a product binomial likelihood as follows:

$$L(q_1, q_2, \dots) = \prod_{j=1}^{N} \binom{m}{y_j} q_j^{y_j} (1 - q_j)^{m - y_j}. \tag{1}$$

We denote by N_r the number of variants appearing exactly r times ($r \geq 0$) in the sample of m tumors (i.e., N_r is the *frequency of frequency* r).

Good-Turing estimation of variant probabilities q_j's

The maximum likelihood estimator y_j/m of q_j is unsuitable for our problem as it places zero probability mass on hitherto unseen variants. Instead, we consider the Good-Turing estimator[1] of word frequencies used in computational linguistic applications. In the original Good-Turing estimation, the variants (words) themselves are the sampling units drawn at random from a large but finite population, resulting in a multinomial likelihood of the variant probabilities (of the form $\binom{\sum_j y_j}{y_1, \dots, y_N} \prod_{j=1}^{N} q_j^{y_j}$), instead of the product binomial likelihood as described in (1). We adapt the original Good-Turing estimator, and obtain an analogous version for our current sampling model, by deriving the estimator from a non-parametric empirical Bayes perspective[2,3] under the current likelihood. Let the q_j's be a priori independent with common prior distribution F on $[0, 1]$. In an empirical Bayes framework, the prior F is itself estimated from the data and the resulting posterior mean is used as point estimate of p_j. Under a general (non-parametric) prior F, the posterior mean of q_j conditional on $y_j = r$, is given by:

$$E(q_j \mid y_j = r) = \frac{\int_0^1 q \binom{m}{r} q^r (1-q)^{m-r} \, dF(q)}{\int_0^1 \binom{m}{r} q^r (1-q)^{m-r} \, dF(q)}.$$

Using the identity

$$q \binom{m}{r} q^r (1-q)^{m-r} = \frac{r+1}{m+1} \binom{m+1}{r+1} q^{r+1} (1-q)^{(m+1)-(r+1)}$$

in the expression for the posterior mean above, we obtain

$$\begin{aligned} E(q_j \mid y_j = r) &= \frac{r+1}{m+1} \frac{\int_0^1 \binom{m+1}{r+1} \, q^{r+1} (1-q)^{(m+1)-(r+1)} \, dF(q)}{\int_0^1 \binom{m}{r} \, q^r (1-q)^{m-r} \, dF(q)} \\ &= \frac{r+1}{m+1} \frac{p_{m+1}(r+1)}{p_m(r)}. \end{aligned} \tag{2}$$

Here, for a fixed cohort size m, and for $r = 0, 1, \ldots,$ $p_m(r)$ denotes the marginal density (mass) of r: $\int_0^1 \binom{m}{r} q^r (1-q)^{m-r}\, dF(q)$, i.e., the marginal probability of a variant frequency being exactly equal to r. An empirical Bayes point estimate of q_j is therefore obtained by using an estimate of the ratio of marginal densities $p_{m+1}(r+1)/p_m(r)$ in (2). In the original Good-Turing estimation[1], the ratio $p_{m+1}(r+1)/p_m(r)$ is estimated by the ratio of empirical frequencies (of frequencies) N_{r+1}/N_r. However, the resulting estimates are often unstable, as many N_r's for different (usually large) r's can be zero, thus making the estimation of $E(q_j \mid y_j = r)$ problematic. To overcome this instability, a smoothing of the raw N_r values is necessary[1] (see next section).

Smoothing raw N_r values produces a regularized value $S(N_r)$ for each r; one then proceeds to Good-Turing estimation by replacing $p_{m+1}(r+1)/p_m(r)$ with $S(N_{r+1})/S(N_r)$ to obtain the following Good-Turing estimate of q_j:

$$\hat{q}_j^{GT} = \frac{y_j + 1}{m+1}\, \frac{S(N_{y_j+1})}{S(N_{y_j})}. \tag{3}$$

This provides a straightforward formula for the probability estimate of a variant j with sample frequency $y_j \geq 1$. However, use of this formula for estimating the probability for a variant that has not yet been observed requires knowledge of N_0, the total number of unseen variants, an unknown quantity. We circumvent this problem by considering the probability of encountering at least one new variant (i.e., the *total probability of unseen variants*) in a new tumor outside of the training sample, which, as we show in the following, can be estimated without any knowledge of N_0.

The probability of encountering *at least one* new variant on a new tumor is given by

$$\pi_0 = 1 - \prod_{\{j : y_j = 0\}} (1 - q_j). \tag{4}$$

The Good-Turing estimated probability of each unseen variant, i.e., each q_j such that $y_j = 0$, is obtained from (3) as (note that $S(N_r) \approx N_r$ for small r, as described in the following section)

$$\tilde{Q}_0 = \frac{1}{m+1}\, \frac{N_1}{N_0},$$

and consequently, an estimate of π_0 is given by

$$\hat{\pi}_0 = 1 - \left(1 - \frac{1}{m+1}\, \frac{N_1}{N_0}\right)^{N_0}.$$

By assuming that N_0, the total number of unseen mutations, is (moderately) large, the limiting definition of the exponential function yields

$$\hat{\pi}_0 \approx 1 - \exp\left[-\frac{N_1}{m+1}\right]. \tag{5}$$

The above quantity only requires the number of singletons (N_1) and the sample size (m), and thus can be computed on any dataset, without requiring any knowledge of the total number of unseen variants (N_0).

Smoothing N_r's in the Good-Turing estimation

We note that smoothing of the raw N_r values entails regularization of observed frequencies of both large and small r's. First, for large r, the raw N_r's are often zero (for example, $N_{r_{\max}+1}$ is always zero, where $r_{\max}$ denotes the maximum observed frequency r). To address this issue, several smoothing techniques have been suggested; in this study we use a simple yet powerful smoothing algorithm based on simple linear regression of $\log N_r$ on $\log r$, originally proposed in Gale and Sampson[4]. This method replaces N_r by the quantity obtained from the regression line when r is large, but keeps N_r intact when r is small (the threshold determining which values of r are *large* is also provided), resulting in an adjustment $S(N_r)$ of N_r (with $S(N_r) = N_r$ for small r).

Second, in some cases (e.g., when considering tissue and gene specific mutations), N_r's are zero for small r's (more specifically, $r = 1$ and 2) and not many positive N_r's are available. Direct implementation of Gale and Sampson smoothing is problematic in such cases. Instead, we first use the following simple imputation strategy for zero N_1 and/or N_2. If there are multiple (say K) populations of tumors (e.g., those specific to different cancer types) under consideration with sample cohort sizes $m_1, \ldots, m_K$, then we replace zero N_1 and N_2 values in the k-th population with $(m_k + 1)/(\sum_{k'=1}^{K} m_{k'} + 1)$, $k = 1, \ldots, K$. This ensures that the estimated total probability of unseen variants (5) remains the same across all populations with no singleton variant in the sample. If, however, only one population of tumors is under consideration (e.g., while considering pan-cancer data), then zero N_1 and N_2 values are simply replaced by 1. This imputation is then followed by an application of Gale and Sampson smoothing.

Estimating the number of unseen variants in a future cohort: Smoothed Good-Toulmin estimator

The estimation of the number of hitherto unseen variants is analogous to the species richness problem in ecology, where the aim is to estimate the total number of unseen species present in a closed population. The most popular statistical model used for this task is the extrapolation approach first introduced in Fisher et al.[5]. Here, one first observes the incidences of variants ("species") in m tumors, and then based on the observed distribution, one considers the problem of estimating/predicting the number of new variants ("species") $\Delta(t)$ that would be observed if mt additional tumors outside the original sample were sequenced. In this study, we focus on the smoothed Good-Toulmin estimator[6,7,8] of $\Delta(t)$, which was originally proposed for $t \leq 1$ in Good and Toulmin[6] under a multinomial framework as discussed earlier. Later, Efron and Thisted[7] modified the estimator, provided an emprirical Bayes justification, and noted the empirical prediction for $\Delta(t)$ for some $t > 1$, albeit without provable guarantees. Recently, Orlitsky et al.[8] have shown that the estimator is also applicable in the context of the product binomial model considered in this study, and provides consistent estimation of $\Delta(t)$ for $t \propto \log m$.

The smoothed Good-Toulmin estimator of $\Delta(t)$ is given by the formula:

$$\hat{\Delta}^{\mathrm{SGT}}(t) = \begin{cases} \sum_{r=1}^{\infty} (-1)^{r+1} t^r N_r & \text{if } t \leq 1 \\ \sum_{r=1}^{\infty} (-1)^{r+1} t^r P\left[\mathrm{Binomial}\left(k(t), \theta(t)\right) \geq r\right] N_r & \text{if } t > 1 \end{cases} \tag{6}$$

with (approximate) variance[7]

$$\mathrm{var}\hat{\Delta}^{\mathrm{SGT}}(t) \approx \begin{cases} \sum_{r=1}^{\infty} t^{2r} N_r & \text{if } t \leq 1 \\ \sum_{r=1}^{\infty} t^{2r} \left(P\left[\mathrm{Binomial}\left(k(t), \theta(t)\right) \geq r\right]\right)^2 N_r & \text{if } t > 1. \end{cases}$$

Here $k(t)$ and $\theta(t)$ are tuning parameters that depend on both m and t.

Orlitsky et al.[8] suggest using $k(t) = \lfloor 0.5 \log_2(mt^2/(t-1)) \rfloor$ with $\theta(t) = (t+1)^{-1}$, or better (in terms of worst-case normalized mean square error), $k(t) = \lfloor 0.5 \log_3(mt^2/(t-1)) \rfloor$ with $\theta(t) = 2(t+2)^{-1}$, where $\lfloor x \rfloor$ denotes the largest integer contained in a real number x. Under these choices, $\hat{\Delta}^{\mathrm{SGT}}(t)$ is shown to have a worst-case normalized mean square error of order $O(m^{-1/t})$, and thus it provably predicts $\Delta(t)$ for any $t \propto \log m$.

Estimating probability of encountering co-occurring and/or mutually exclusive paired gene mutations

The Good-Turing strategy (2) can also be used to find the probability of encountering co-mutations (or mutual exclusivities) of two specific genes in a randomly chosen tumor. Let us label the possible gene pairs as $1, \ldots, M = \binom{G}{2}$, where G denotes the total number of genes under consideration. Note that G is known, and hence the total number of pairs $M = \binom{G}{2}$ is also known. This makes estimation of the probabilities of encountering hitherto unseen gene co-mutations (or mutual exclusivities) simpler than that of the unseen variants. The co-mutation (mutual exclusivity) frequency u_j of the j-th gene-pair, under a product binomial model similar to (1) for all $j = 1, \ldots, M$, provide analogous Good-Turing estimates of the probability of observing specific gene co-mutations (including the hitherto unobserved ones). The formulas are omitted for brevity.

Determining association between a specific mutation (or co-mutation, or mutual-exclusivity) and the tissue type of the tumor

The normalized mutual information[9] (NMI) criterion provides a rigorous way of quantifying the association between the probability of occurrence of a specific variant (or a co-mutating or mutually exclusive gene-pair) and the set of tissue types evaluated. Let x_j denote the presence (1) or absence (0) of the j-th variant (or j-th gene-pair) in a tumor, and let C denote the type of tissue associated with the tumor, $C = 1, \ldots, K$. The normalized mutual information between x_j and C is given by:

$$\text{NMI}(x_j, C) = \frac{MI(x_j, C)}{\sqrt{H(x_j)\ H(C)}}$$

where $\text{MI}(x_j, C)$ is the mutual information[10] between x_j and C defined as

$$\begin{aligned}\text{MI}(x_j, C) &= \sum_{x=0}^{1}\sum_{k=1}^{K} P(x_j = x, C = k) \log \frac{P(x_j = x, C = k)}{P(x_j = x)P(C = k)} \\ &= \sum_{x=0}^{1}\sum_{k=1}^{K} P(x_j = x \mid C = k)P(C = k) \log \frac{P(x_j = x \mid C = k)}{\sum_{k=1}^{K} P(x_j = x \mid C = k)P(C = k)}\end{aligned}$$

and $H(x_j)$ and $H(c)$ denote the (marginal) Shannon entropies of x_j and c respectively, defined as

$$
\begin{aligned}
H(x_j) &= \sum_{x=0}^{1} \log P(x_j = x)\ P(x_j = x) \\
&= \sum_{x=0}^{1} \sum_{k=1}^{K} \log \left(\sum_{k=1}^{K} P(x_j = x \mid c = k) P(c = k) \right) P(x_j = x \mid c = k)\ P(c = k)
\end{aligned}
$$

and

$$H(c) = \sum_{k=1}^{K} \log P(c = k)\ P(c = k).$$

In the above formulas, the tissue-type specific variant (or co-mutating, or mutually-exclusive gene pair) probabilities $P(x_j = 1 \mid C = k)$ are estimated using the Good-Turing method, and the tissue type probabilities $P(C = k)$ are estimated by the proportion $m_k / \sum_{i=1}^{K} m_i$, where, as before, m_k denotes the number of tumors in the cohort coming from the k-th tissue type. The NMI values plotted in Figures 3a, 3b, 3c, 5a and 5b are computed using this formula.

A related quantity of interest is the normalized mutual information between x_j and the binary event $C_k = \mathbb{1}\{C = k\}$, $k = 1, \ldots, K$, where $\mathbb{1}(\cdot)$ denotes the indicator function. We use this quantity in Supplementary figures 6a and 6b to evaluate the extent to which individual co-mutating and mutually exclusive paired gene mutations are preferred within specific tumor types.

$$\text{NMI}(x_j, C_k) = \frac{MI(x_j, C_k)}{\sqrt{H(x_j)\ H(C_k)}}$$

where

$$\text{MI}(x_j, C_k) = \sum_{x=0}^{1} \sum_{\alpha=0}^{1} P(x_j = x, C_k = \alpha) \log \frac{P(x_j = x, C_k = \alpha)}{P(x_j = x) P(C_k = \alpha)}.$$

The identities

(i) $P(C_k = 1) = P(C = k)$

(ii) $P(x_j = x, C_k = 1) = P(x_j = x \mid C = k) P(C = k)$

(iii) $P(x_j = x) = \sum_{k=1}^{K} P(x_j = x \mid C = k) P(C = k)$, and

(iv) $P(x_j = x, C_k = 0) = P(x_j = x) - P(x_j = x, C_k = 1)$

for $x = 0, 1$, along with the Good-Turing estimates of $P(x_j = x \mid C = k)$ and the relative frequency estimates of $P(C = k)$ provide estimates of the component quantities in $\mathrm{MI}(x_j, C_k)$. $H(C_k)$ is defined as

$$H(C_k) = \sum_{\alpha=0}^{1} \log P(C_k = \alpha)\ P(C_k = \alpha)$$

and is estimated by plugging in the relative proportion $m_k / \sum_{k=1}^{K} m_k$ of tumors with k-th tissue type for $P(C_k = 1)$ in the above formula.

The NMI is a real number between 0 and 1 with a zero NMI value indicating independence. Thus, $\mathrm{NMI}(x_j, C)$ (or $\mathrm{NMI}(x_j, C_k)$) quantifies the dependence between the occurrence (co-occurrence or mutually exclusive occurrence) of j-th variant (gene pair) x_j, and the tissue type C (or the tissue type C being k, i.e., the event $\{C = k\}$) for a randomly chosen tumor, with a high $\mathrm{NMI}(x_j, C)$ (or $\mathrm{NMI}(x_j, C_k)$) indicating a high dependence.

Null reference distribution for Mutual Information

To determine the strength of the variant-tissue specificities "observed" in the training data (as quantified by NMI), we compare the computed NMI values to reference values obtained by simulating results under the assumption that there is no tissue specificity, i.e. variants (or co-mutating gene pairs or mutually exclusive gene-pairs) occur independent of tissue type. For this, we generate multiple (1000 in this study) instances of random variant-tissue allocations, obtained by randomly permuting the tissue-type ("cancer label") associated with each tumor in the training data. Note that this permutation keeps the sample sizes of tissue types unaltered. For each permutation, (gene and tissue specific) Good-Turing variant probability estimates are computed, which are then subsequently used to calculate the normalized mutual information between the occurrence of a variant and the type of the tissue. These (permutation) NMI values collectively constitute the null reference distribution. In Figure 3c, the orange histogram summarizes this null distribution, while the blue histogram corresponds to the observed values of NMI between the occurrence of at least one new variant in a cancer gene (in the oncoKB list) and the type of the tissue, with 0.01 being the 95-th percentile of the NMI values under null reference distribution.

Mutation signature analysis

The mutation signature analysis was performed by pre-specifying mutational signatures in advance using the algorithm outlined in Zehir et al.[11], and the implementation available https://github.com/mskcc/mutation-signatures. For each tumor, we obtained the dominant single base substitution number according to the Sanger COSMIC mutation signature annotation[12], and categorized the tumor into one of six categories: non-hypermutated, APOBEC (apolipoprotein B mRNA editing enzyme catalytic polypeptide-like), Smoking-associated, MMR (mismatch repair), UV (ultraviolet) and POLE (DNA Polymerase Epsilon, Catalytic Subunit).

Supplementary Tables

Supplementary Table 1: Cancer categories and cohort sizes in TCGA data

Cancer Code	Disease	No. of tumors
ACC	Adrenocortical carcinoma	92
BLCA	Bladder Urothelial Carcinoma	411
BRCA	Breast invasive carcinoma	1025
CESC	Cervical squamous cell carcinoma and endocervical adenocarcinoma	291
CHOL	Cholangiocarcinoma	36
COADREAD	Colorectal adenocarcinoma	559
DLBC	Lymphoid Neoplasm Diffuse Large B-cell Lymphoma	37
ESCA	Esophageal carcinoma	185
GBM	Glioblastoma multiforme	401
HNSC	Head and Neck squamous cell carcinoma	509
KICH	Kidney Chromophobe	66
KIRC	Kidney renal clear cell carcinoma	370
KIRP	Kidney renal papillary cell carcinoma	282
LAML	Acute Myeloid Leukemia	136
LGG	Brain Lower Grade Glioma	525
LIHC	Liver hepatocellular carcinoma	365
LUAD	Lung adenocarcinoma	568
LUSC	Lung squamous cell carcinoma	485
MESO	Mesothelioma	81
OV	Ovarian serous cystadenocarcinoma	412
PAAD	Pancreatic adenocarcinoma	176
PCPG	Pheochromocytoma and Paraganglioma	183
PRAD	Prostate adenocarcinoma	495
SARC	Sarcoma	239
SKCM	Skin Cutaneous Melanoma	468
STAD	Stomach adenocarcinoma	438
TGCT	Testicular Germ Cell Tumors	150
THCA	Thyroid carcinoma	499
THYM	Thymoma	123
UCEC	Uterine Corpus Endometrial Carcinoma	531
UCS	Uterine Carcinosarcoma	57
UVM	Uveal Melanoma	80

Supplementary Table 2: Cancer categories and cohort sizes in MSK-IMPACT data

Cancer Type	No. of tumors
Appendiceal Cancer	73
Bladder Cancer	392
Bone Cancer	71
Breast Cancer	1116
Cancer of Unknown Primary	161
Cervical Cancer	43
Colorectal Cancer	969
Endometrial Cancer	205
Esophagogastric Cancer	291
Gastrointestinal Neuroendocrine Tumor	35
Gastrointestinal Stromal Tumor	98
Germ Cell Tumor	182
Glioma	487
Head and Neck Cancer	154
Hepatobiliary Cancer	296
Mature B-Cell Neoplasms	123
Melanoma	339
Mesothelioma	70
Non-Small Cell Lung Cancer	1473
Ovarian Cancer	198
Pancreatic Cancer	463
Peripheral Nervous System	42
Prostate Cancer	505
Renal Cell Carcinoma	269
Salivary Gland Cancer	77
Skin Cancer, Non-Melanoma	110
Small Bowel Cancer	34
Small Cell Lung Cancer	81
Soft Tissue Sarcoma	272
Thyroid Cancer	203
Uterine Sarcoma	69

Supplementary Figures

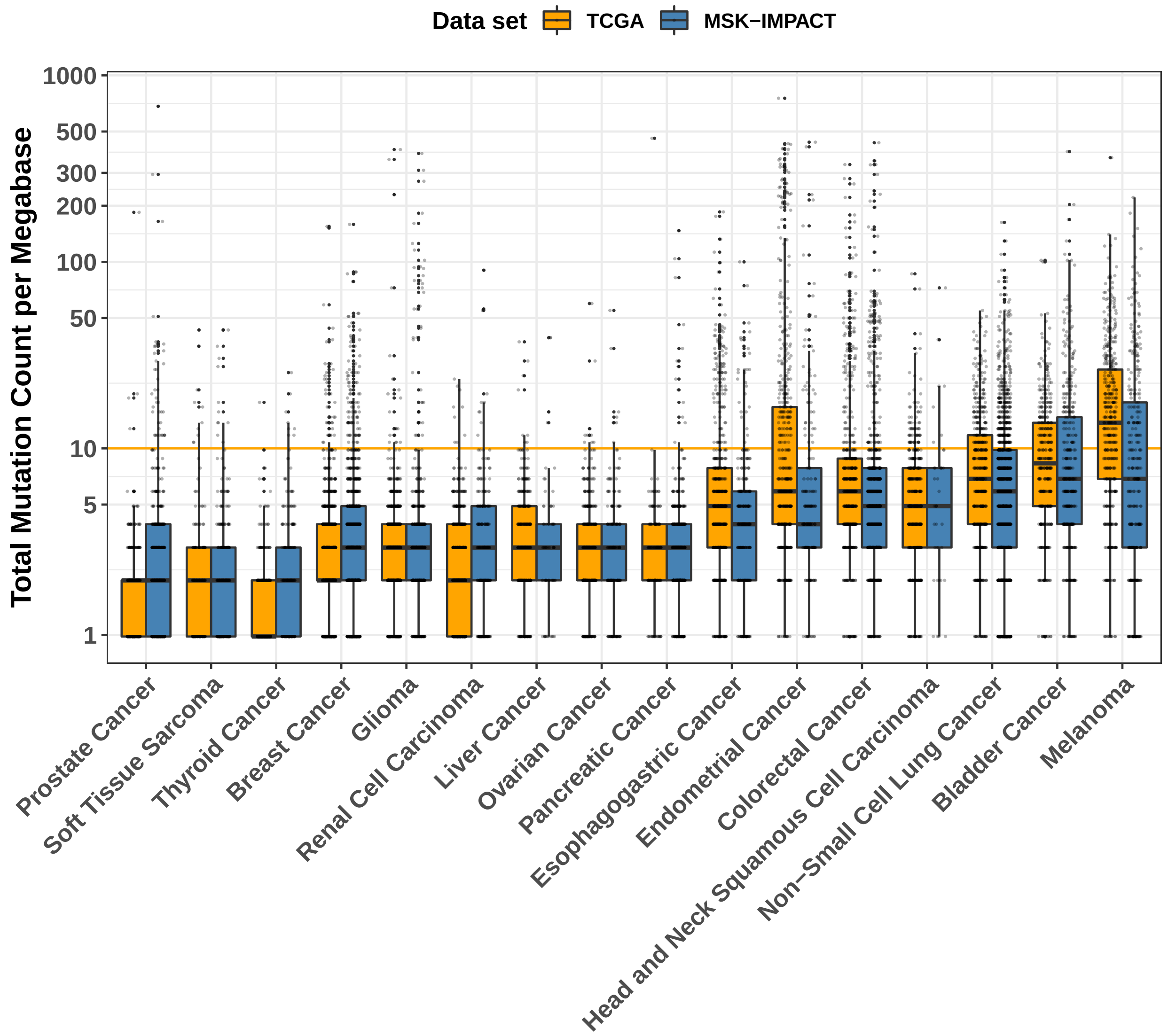

Supplementary Figure 1: Distributions of total mutation burden in tumors among the 410 MSK-IMPACT genes are largely similar in the TCGA data set (orange box plots) and in the MSK-IMPACT data set (blue box plots) across common cancer types.

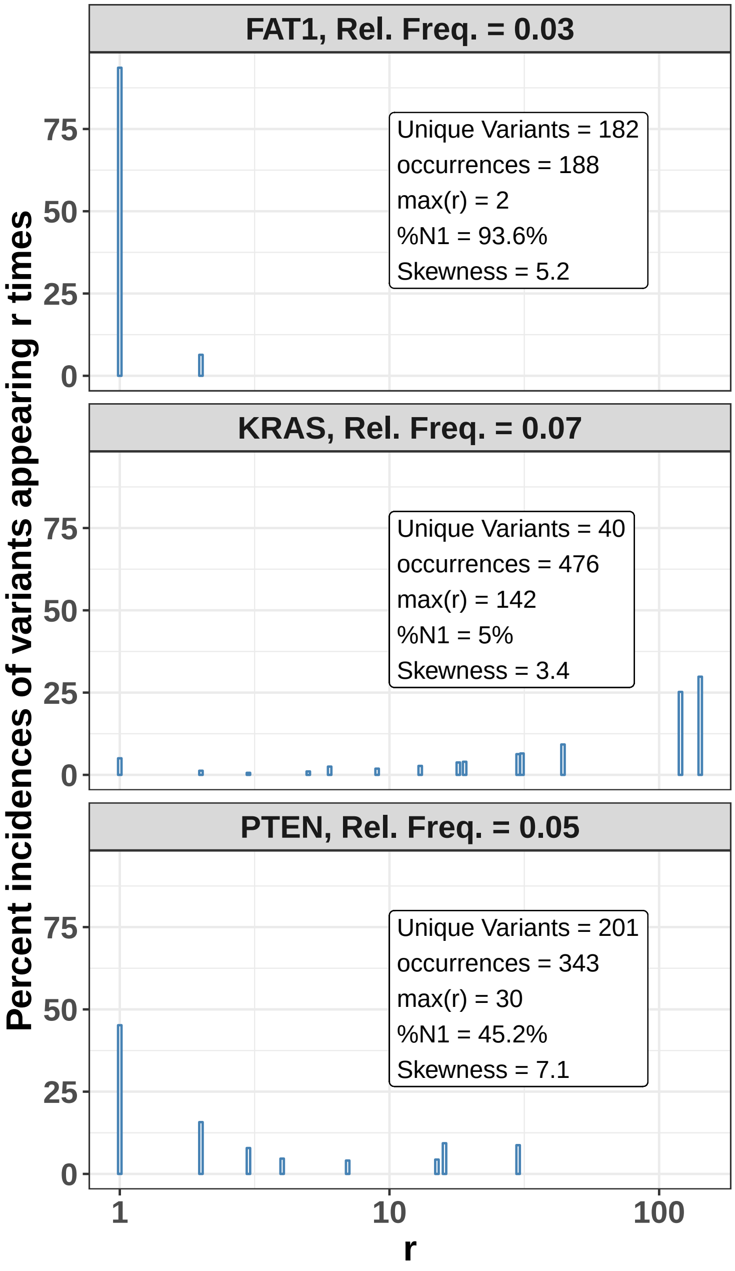

KRAS

r	N_r
1	24
2	3
3	1
5	1
6	2
9	1
13	1
18	1
19	1
30	1
31	1
44	1
120	1
142	1

PTEN

r	N_r
1	155
2	27
3	9
4	4
7	2
15	1
16	2
30	1

FAT1

r	N_r
1	176
2	6

Supplementary Figure 2: (Pan-cancer) variants from different genes show different frequency r, N_r distributions, as illustrated by the three cancer genes (in OncoKB list) *KRAS*, *FAT1* and *PTEN*.

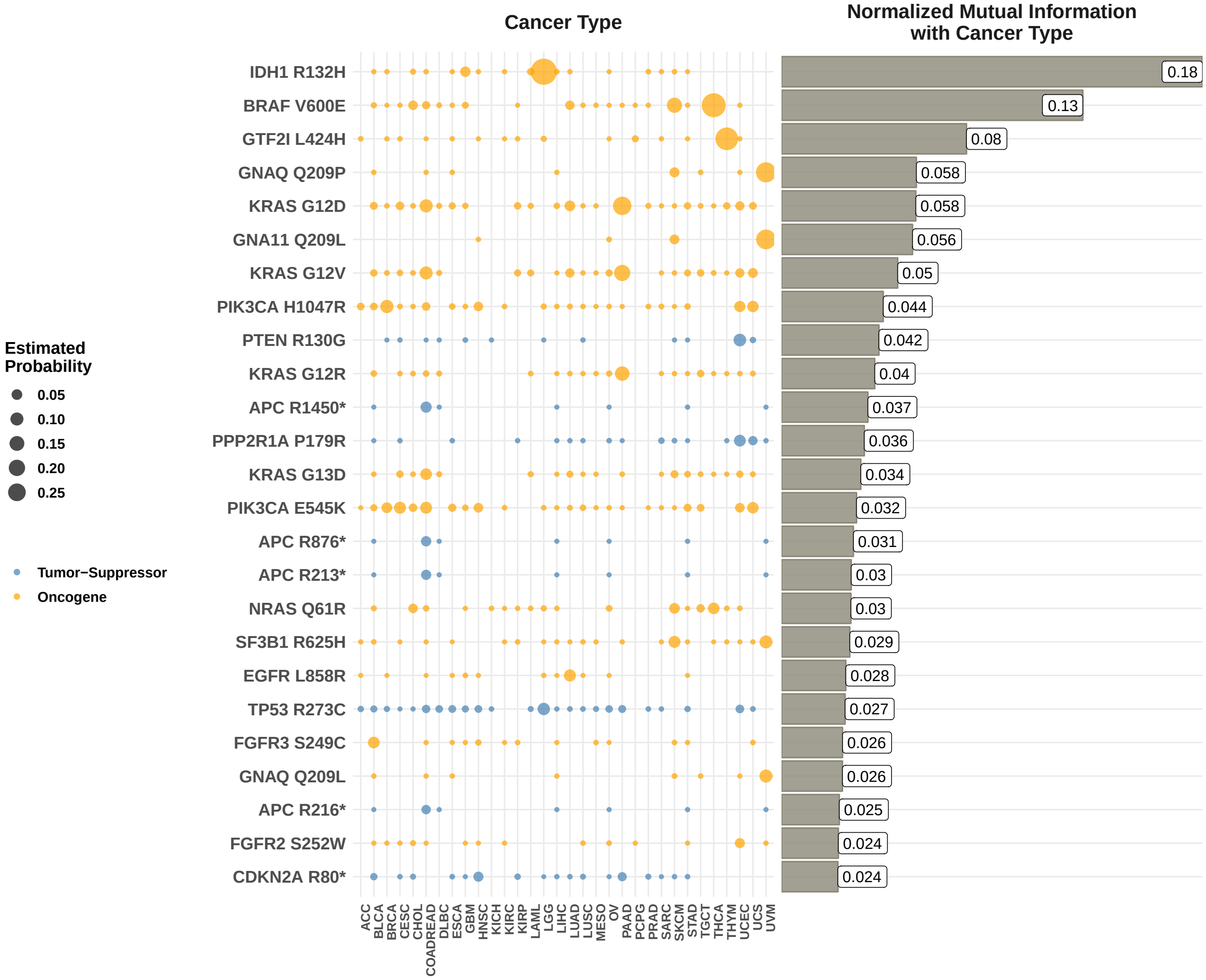

Supplementary Figure 3: Probability of encountering the top 25 observed variants (ranked by NMI) for different tissue types, and their NMI with the type of tissue.

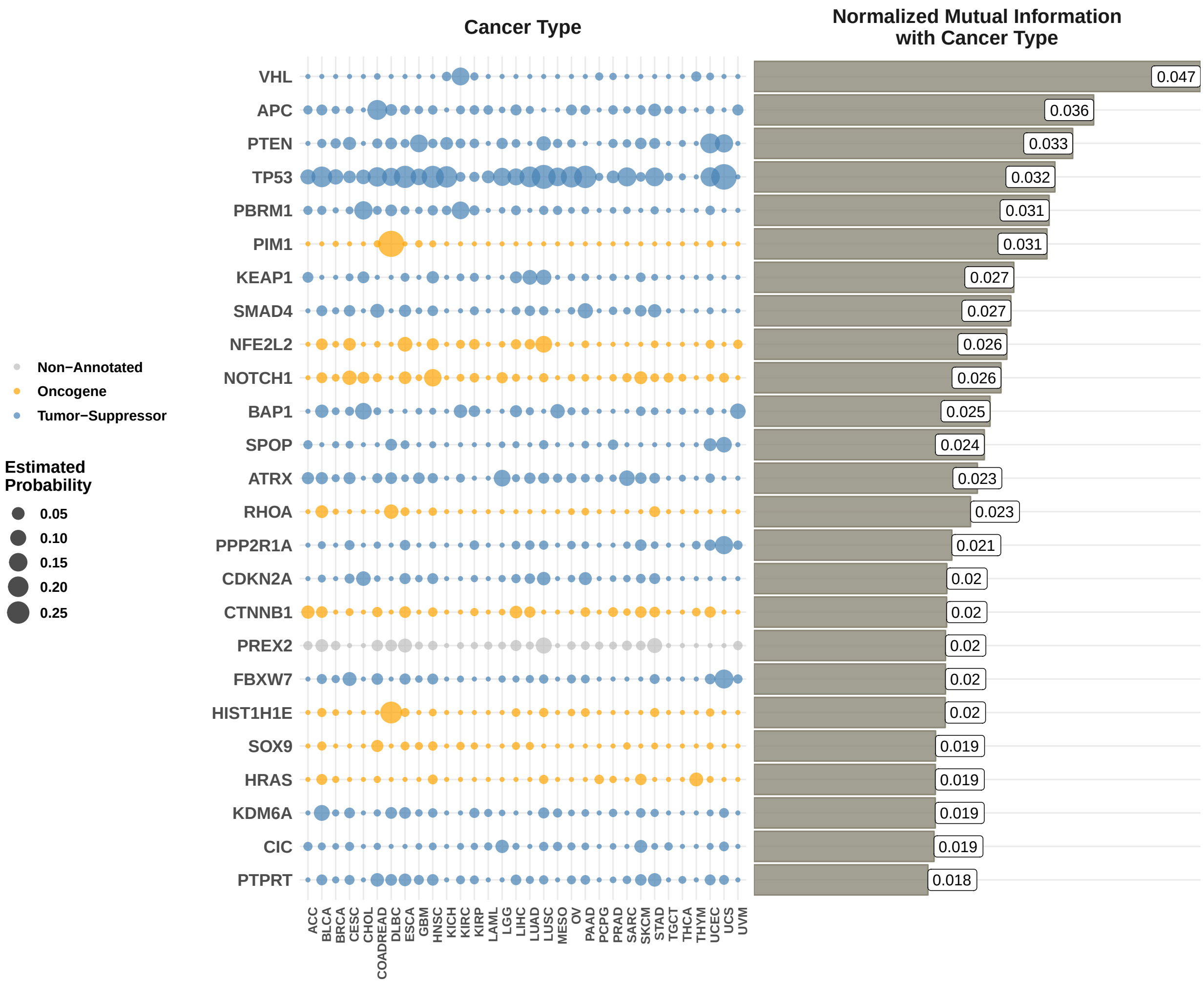

Supplementary Figure 4: Probability of encountering at least one new variant in a new tumor for top 25 genes (ranked by NMI) for different tissue types, and their NMI with the type of tissue.

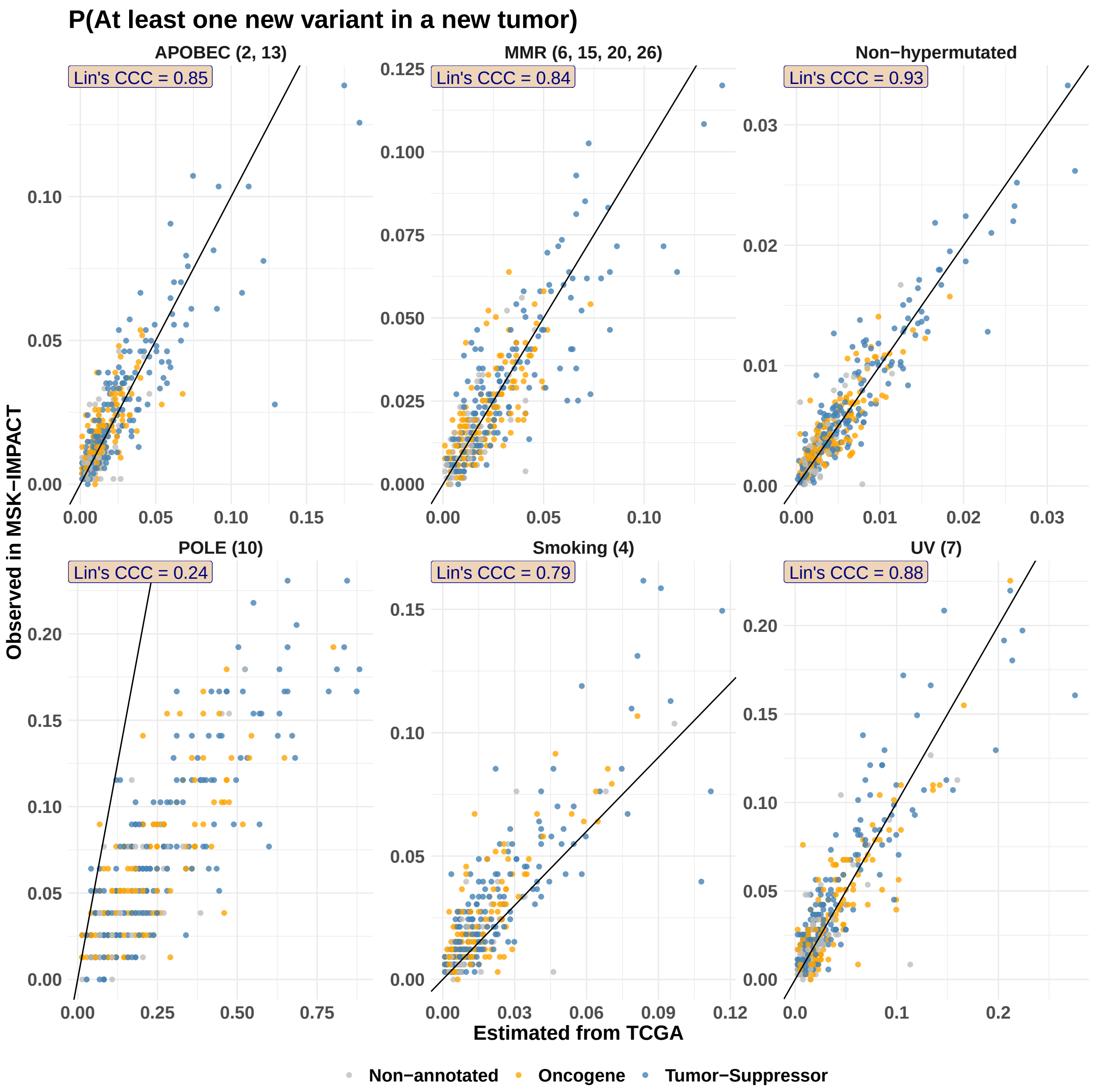

Supplementary Figure 5: The TCGA estimated probability, plotted against the observed relative frequency of at least one new variant in a new tumor in MSK-IMPACT data for *all* MSK-IMPACT genes, plotted separately for the six tumor subgroups. Oncogenes are shown in orange, tumor-suppressor genes are in steelblue, and non-annotated (in OncoKB list) genes are shown in gray.

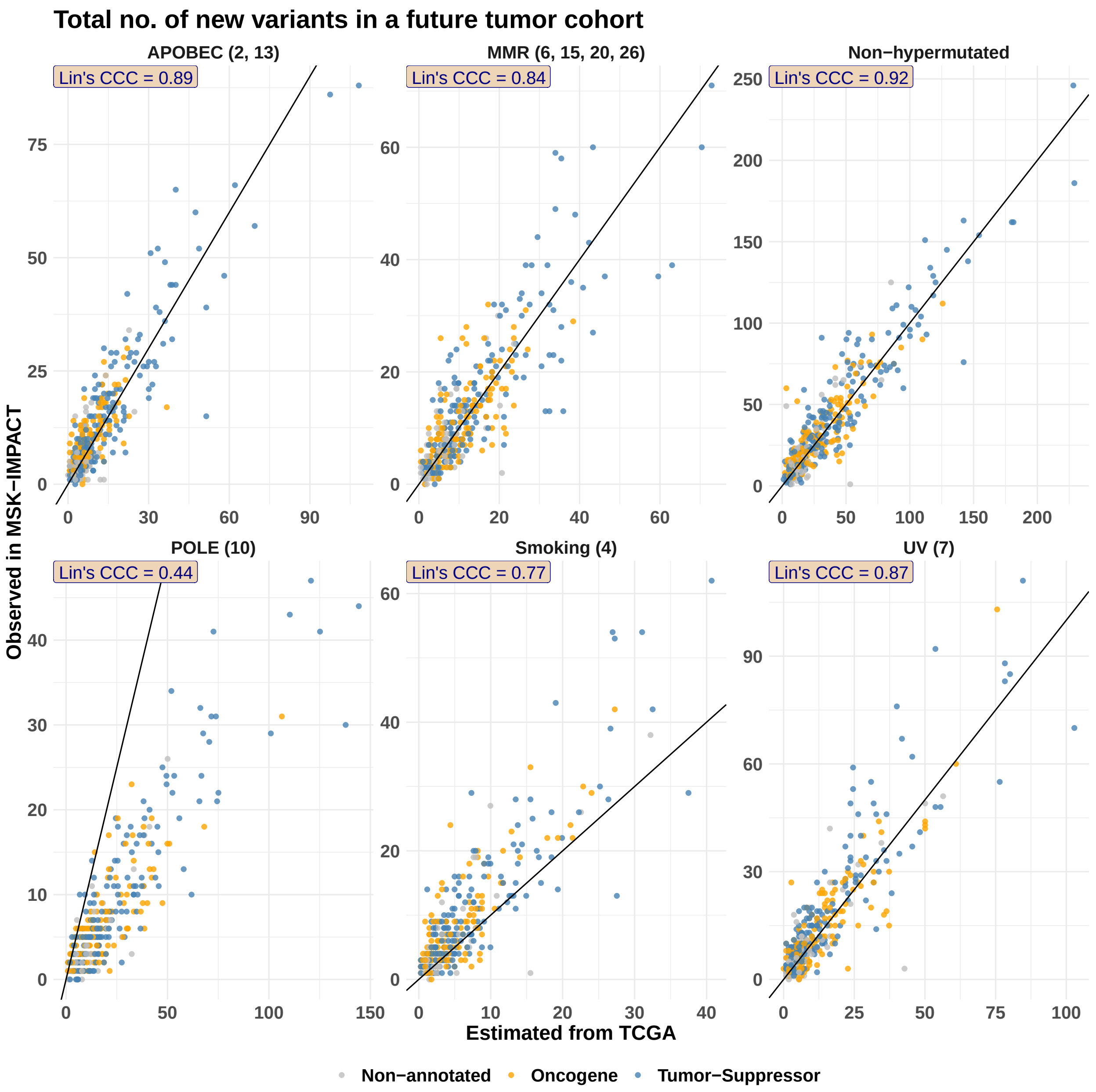

Supplementary Figure 6: The TCGA predicted total number of new variants to be observed a future tumor cohort, plotted against the observed number of new variants per tumor in MSK-IMPACT data for *all* MSK-IMPACT genes, plotted separately for the six tumor subgroups. Oncogenes are shown in orange, tumor-suppressor genes are in steelblue, and non-annotated (in OncoKB list) genes are shown in gray.

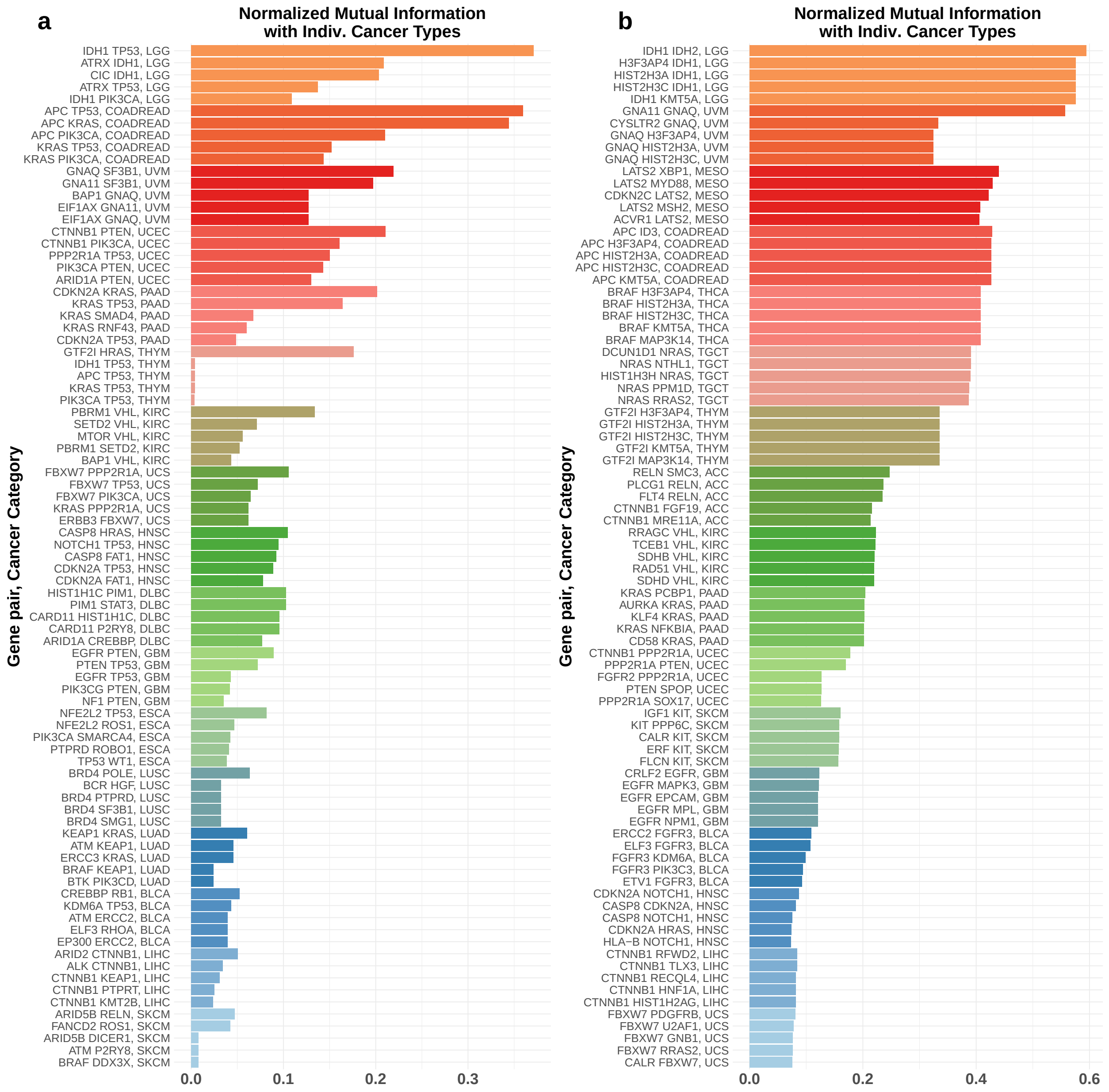

Supplementary Figure 7: Gene co-mutation and mutual exclusivity probabilities are very dependent on the type of cancer. The occurrences of gene co-mutations (Panel – a) and mutual exclusivity (Panel – b) also depend heavily on the specific type of cancer, as evidenced by the variability among the NMI values between the occurrences of specific gene pairs and the binary indicators of individual cancer categories.

Supplementary References

[1] Good, I. J. The population frequencies of species and the estimation of population parameters. *Biometrika* **40**, 237–264 (1953).

[2] Robbins, H. An empirical bayes approach to statistics. In *Proceedings of the Third Berkeley Symposium on Mathematical Statistics and Probability, Volume 1: Contributions to the Theory of Statistics*, 157–163 (University of California Press, Berkeley, Calif., 1956). URL https://projecteuclid.org/euclid.bsmsp/1200501653.

[3] Nadas, A. On turing's formula for word probabilities. *IEEE Transactions on Acoustics, Speech, and Signal Processing* **33**, 1414–1416 (1985).

[4] Gale, W. A. & Sampson, G. Good-turing frequency estimation without tears. *Journal of quantitative linguistics* **2**, 217–237 (1995).

[5] Fisher, R. A., Corbet, A. S. & Williams, C. B. The relation between the number of species and the number of individuals in a random sample of an animal population. *Journal of Animal Ecology* **12**, 42–58 (1943). URL http://www.jstor.org/stable/1411.

[6] Good, I. J. & Toulmin, G. H. The number of new species, and the increase in population coverage, when a sample is increased. *Biometrika* **43**, 45–63 (1956). URL http://dx.doi.org/10.1093/biomet/43.1-2.45.

[7] Efron, B. & Thisted, R. Estimating the number of unsen species: How many words did Shakespeare know? *Biometrika* **63**, 435–447 (1976). URL http://www.jstor.org/stable/2335721.

[8] Orlitsky, A., Suresh, A. T. & Wu, Y. Optimal prediction of the number of unseen species. *Proceedings of the National Academy of Sciences* **113**, 13283–13288 (2016). URL https://www.pnas.org/content/113/47/13283. https://www.pnas.org/content/113/47/13283.full.pdf.

[9] Strehl, A. & Ghosh, J. Cluster ensembles - A knowledge reuse framework for combining multiple partitions. In *Journal of Machine Learning Research* (2003).

[10] Cover, T. M. & Thomas, J. A. *Elements of Information Theory* (2005).

[11] Zehir, A. *et al.* Mutational landscape of metastatic cancer revealed from prospective clinical sequencing of 10,000 patients. *Nature Medicine* (2017). 15334406.

[12] Alexandrov, L. B. *et al.* Signatures of mutational processes in human cancer. *Nature* (2013).
